# Supplementary material for: Leveraging locus-specific epigenetic heterogeneity to improve the performance of blood-based DNA methylation biomarkers
Source: Clin Epigenetics. 2020 Oct 20;12:154. doi: 10.1186/s13148-020-00939-w (PMC7574234; doi:10.1186/s13148-020-00939-w)
Supplement: Supplementary file 1 — Additional file 1. Supplementary figures. [file 13148_2020_939_MOESM1_ESM.pdf]

Supplementary Figure S1

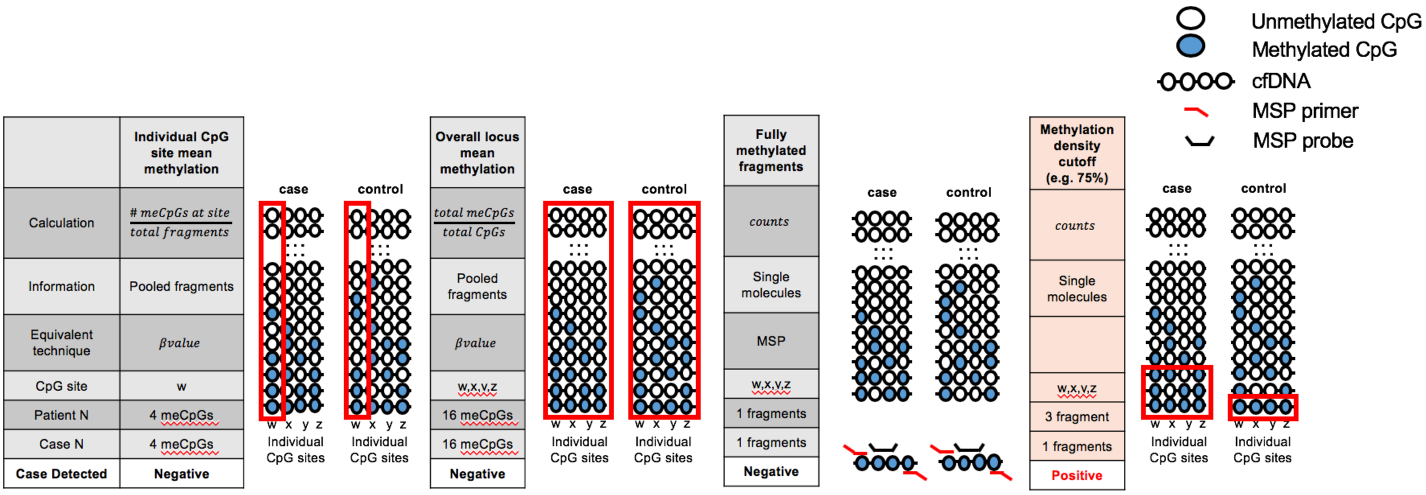

Supplementary Figure S1: Illustration of different approaches for calculating methylation differences between case and control cfDNA epialleles. Case and control are the same examples from Figure 1. Red outlines indicate the read methylation information that is assessed by each approach. Fully-methylated fragments representative of a target queried in methylation-sensitive PCR (MSP) assays. Here, both case and control have equal counts of fully methylated molecules for which the probe can anneal to the target cfDNA (after bisulfite conversion) and are indistinguishable.

Supplementary Figure S2

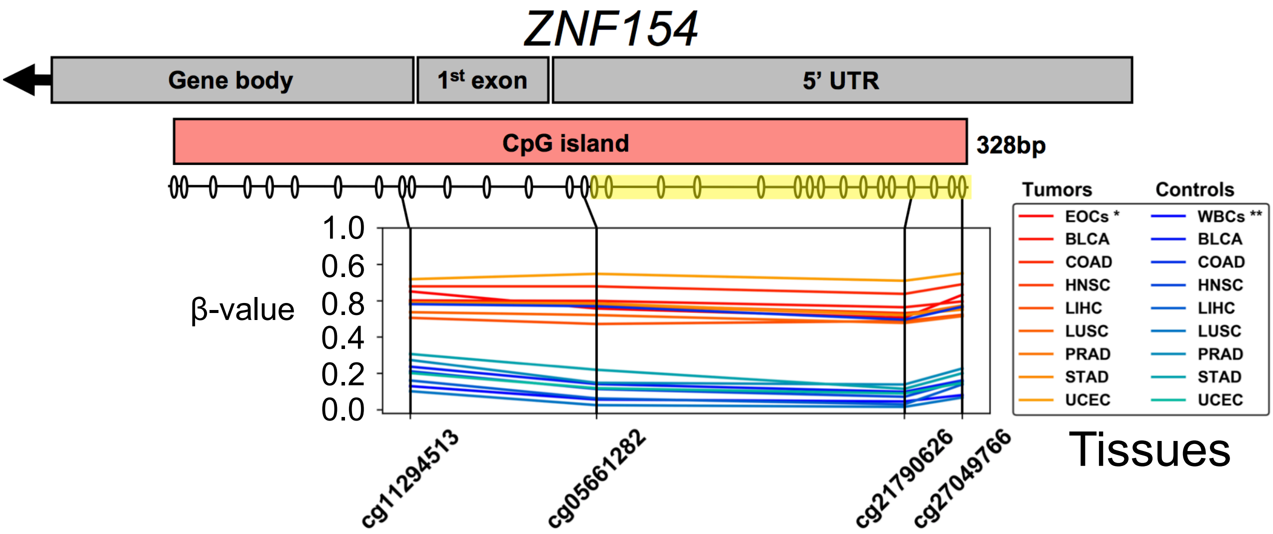

Supplementary Figure S2: Methylation at the *ZNF154* genomic locus. **A**) The *ZNF154* gene is encoded on the reverse strand of Chromosome 19 and contains a 328-bp CpG island (CGI) that extends from the 5'-UTR through into the *ZNF154* gene body itself. Schematic showing β-values at multiple CpG sites determined from Illumina Infinium HumanMethylation450 array data for tumor (*red lines*) and control (*blue lines*) tissues. The target locus assessed in the DREAMing assay is highlighted (*yellow*). Abbreviations: ovals below the CpG island represent CpG positions; EOCs = epithelial ovarian carcinomas (n=221); WBCs = white blood cells; * indicates data taken from Widschwendter *et. al* (8); ** indicates data from Lehne *et. al* (46); remaining 4 letter acronyms correspond to TCGA tissue codes: BLCA = bladder carcinoma (tumors = 412, controls = 21); COAD = colon adenocarcinoma (tumors = 295, controls = 38); HNSC = head-neck squamous cell carcinoma (tumors = 528, controls = 50); LIHC = liver hepatocellular carcinoma (tumors = 377, controls = 50); LUSC = lung squamous cell carcinoma (tumors = 370, controls = 42); PRAD = prostate adenocarcinoma (tumors = 498, controls = 50); STAD = stomach adenocarcinoma (tumors = 395, controls = 2); UCEC = uterine corpus endometrial carcinoma (tumors = 431, controls = 46).

Supplementary Figure S3

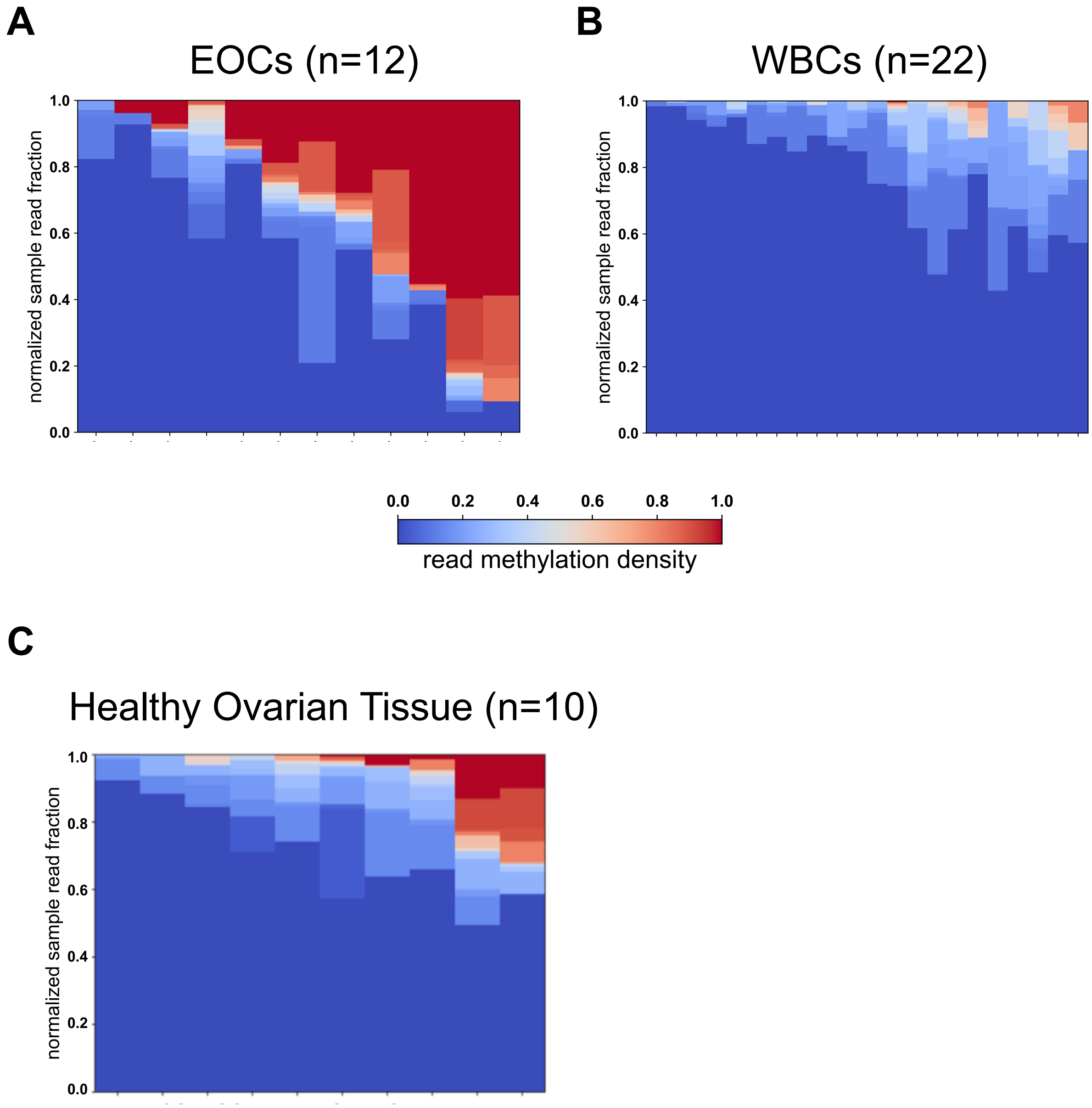

Supplementary Figure S3: Methylation density at the *ZNF154* genomic locus. **A-C**) Heatmaps showing the relative fractions and corresponding methylation density profiles derived from RRBS reads of the *ZNF154* target locus for ovarian carcinomas (n=12), healthy ovarian tissues (n=10), and WBCs (n=22). Abbreviations: WBCs = white blood cells. Data from Widschwendter *et. al* (8).

Supplementary Figure S4

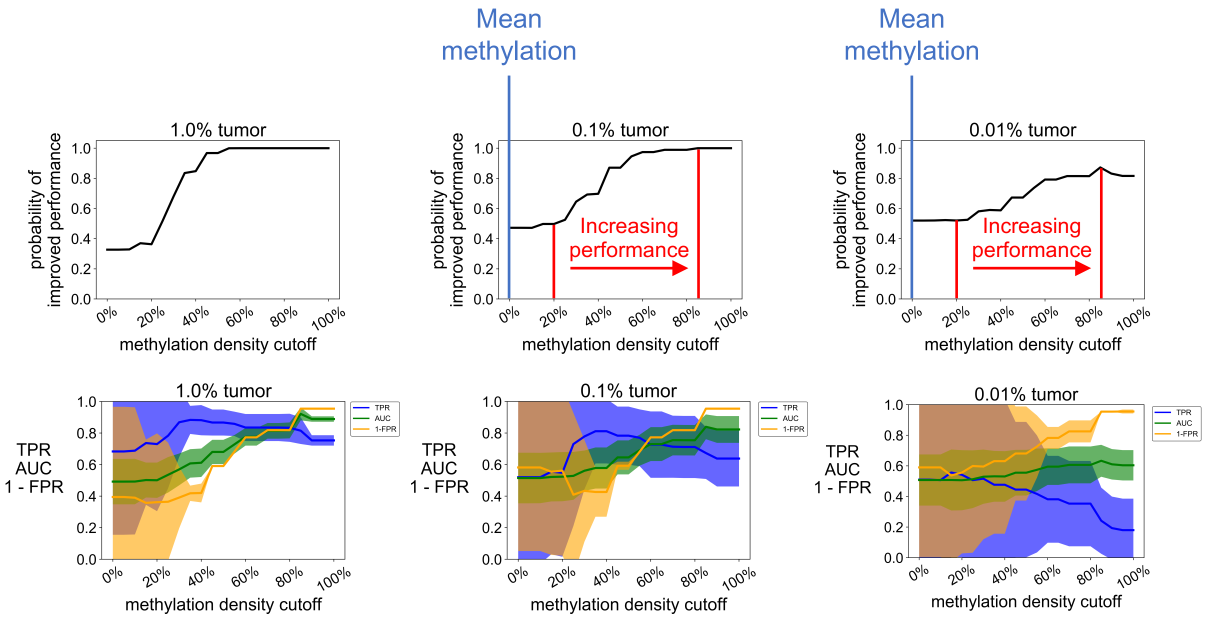

Supplementary Figure S4: Simulated performance of EpiClass using varying admixture ratios of ovarian carcinoma (EOC) to WBC RRBS reads at 10000 reads per sample. Plots showing the probability of achieving a higher AUC using EpiClass compared to the mean locus methylation classifier for each methylation density cutoff at various admixture ratios. For sub-1% tumor fractions, the range of methylation density cutoffs (20%-85%) that result in increasing probability of improved classification performance over mean methylation is indicated between red lines. Mean methylation methylation density cutoff indicated by blue lines. Lower panels show the TPR, 1-FPR, and AUC achieved by using EpiClass at each methylation density cutoff. Solid lines indicate the mean value and shaded regions indicate the 95% confidence interval for 50 iterations of the simulation. Abbreviations: AUC = area under the curve; TPR = true positive rate; FPR = false positive rate.

Supplementary Figure S5

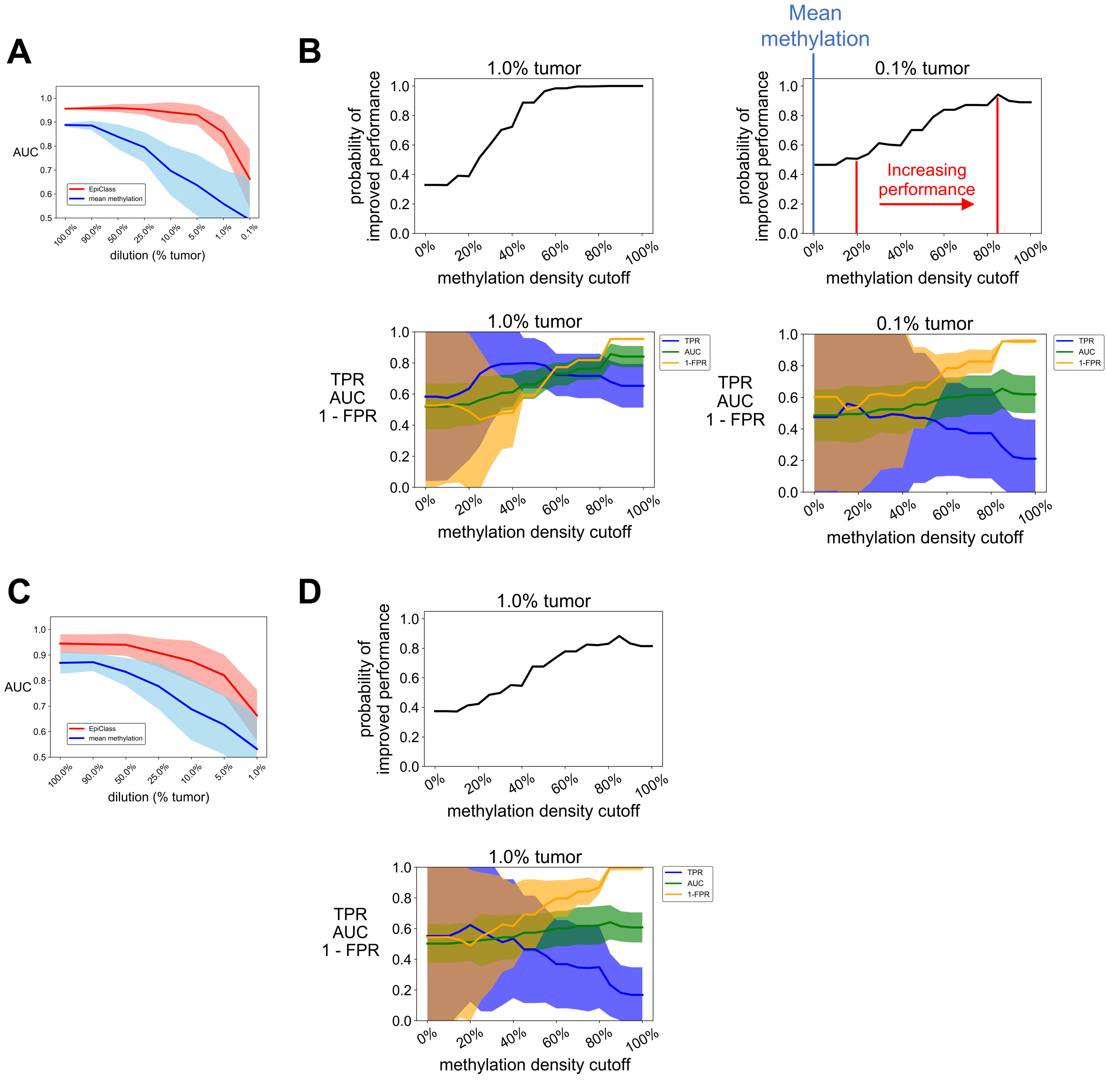

Supplementary Figure S5: Simulated performance of EpiClass using varying admixture ratios of ovarian carcinoma (EOC) to WBC RRBS reads sampled at 100 and 1000 total reads per simulated sample. **A**) The performance of the methylation density binary classifier (EpiClass, *red*) and mean locus methylation classifier (*blue*) at increasing dilutions of EOC RRBS reads in a background of WBC RRBS reads acquired from Widschwendter *et al*. (8) with 1000 total reads sampled. **B**) Plots showing the probability of achieving a higher AUC than the mean locus methylation classifier using EpiClass for each methylation density cutoff at various admixture ratios based on 1000 read per simulated sample. Lower panels show the TPR, 1-FPR, and AUC achieved by using EpiClass at each methylation density cutoff. EOCs (n=12) were randomly paired with a WBC (n=22) sample and RRBS reads were sampled from an EOC-WBC pair to generate a simulated spike-in sample. Simulated samples (n=12) were compared to the original WBC samples (n=22). 50 iterations of the simulation were performed. Solid lines indicate mean values and shaded regions indicate 95% confidence intervals. For sub-1% tumor fractions, the range of methylation density cutoffs (20%-85%) that result in increasing probability of improved classification performance over mean methylation is indicated between red lines. Mean methylation methylation density cutoff indicated by blue lines. **C-D**) Same as A) and B) except with 100 total reads sampled. Abbreviations: EpiClass = methylation density classifier; AUC = area under the curve; TPR = true positive rate; FPR = false positive rate.

Supplementary Figure S6

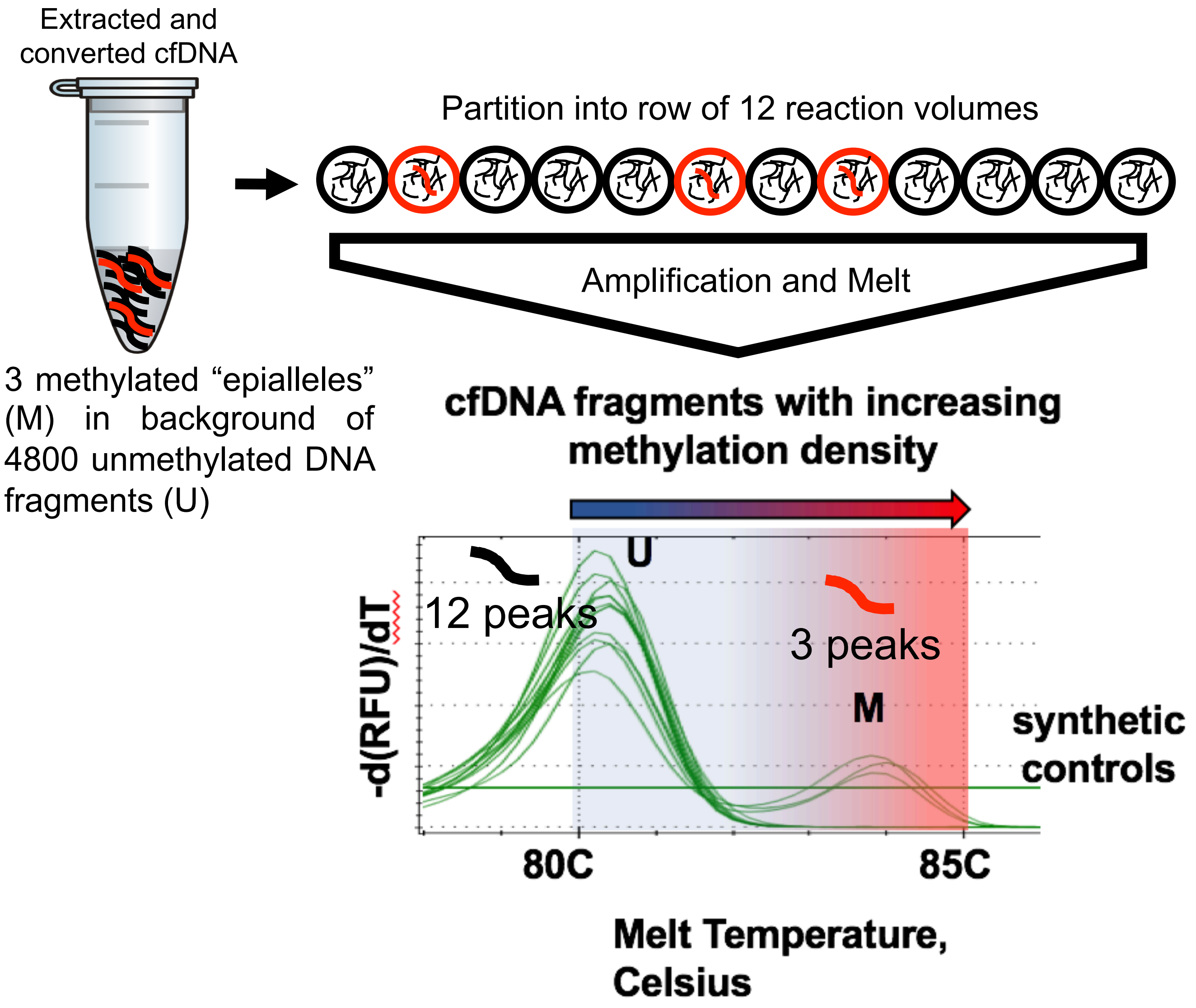

Supplementary Figure S6: Melting traces from DREAMing assay for 12 wells containing in total a mixture of 3 synthetic fully-methylated (*M, red*) and 4800 unmethylated control male genomic DNA (*U, black*) fragments (400 U per well) originating from the *ZNF154* genomic region of interest. The sample is partitioned such that there is no more than 1 methylated epiallele per well (in addition to unmethylated DNA). The 3 rare methylated epialleles are assumed to be distributed among the 12 wells based on a Poissonian distribution. All wells give a melt peak indicative of the presence of unmethylated U background DNA fragments, which all melt at approximately the same temperature. Wells with a methylated epiallele produce a secondary melt peak corresponding to the methylation density of the epiallele. Abbreviations: -d(RFU)/dT = negative derivative of the change in relative fluorescent units. U = unmethylated DNA; M = methylated DNA.

Supplementary Figure S7

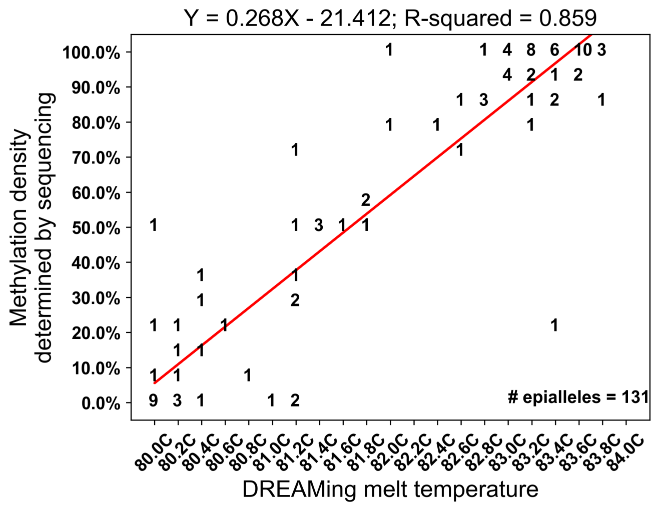

Supplementary Figure S7: *ZNF154* locus methylation density vs. DREAMing melt temperature in cfDNA. DREAMing melt peak temperatures and corresponding methylation density measurements identified via bisulfite sequencing for 131 post-DREAMing epiallele amplicons. The numbers on the plot represent the number of times a detected melt peak had the corresponding melt temperature and produced an amplicon with the corresponding methylation density. The red line denotes the best fit regression line. The linear regression model and R-squared value is shown above the plot.

Supplementary Figure S8

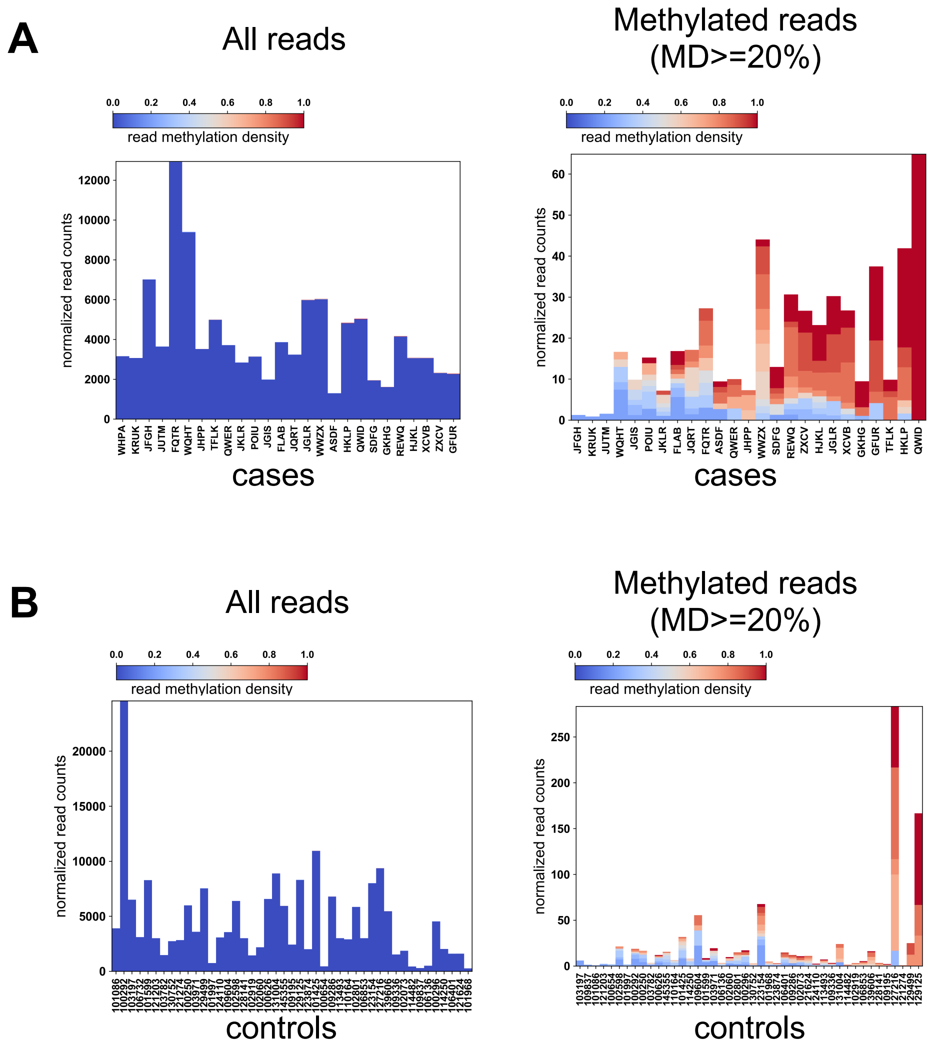

Supplementary Figure S8: Fraction of total reads and methylated reads in case (A) and control (B) samples from the training cohort. Methylated reads are considered epialleles with a methylation density >= 20%. Y-axes indicate the normalized counts of reads (epialleles per mL that were quantified in DREAMing) for each sample. Color bars indicate the methylation density of the quantified reads. MD = “methylation density”. X-axes indicate sample IDs.

Supplementary Figure S9

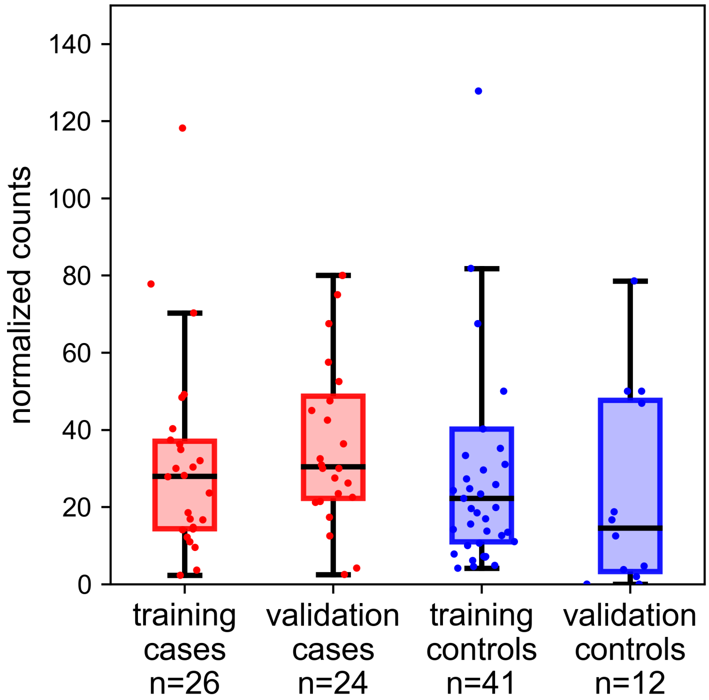

Supplementary Figure S9: Total normalized counts (counts per mL sample plasma) of methylated reads with any methylation in cases and controls from the training and validation sample cohorts. Methylated reads include all reads with a methylation density > 0%. No statistical difference between the sample cohorts; two-sided Wilcoxon rank-sum test: Training cases median counts = 28.0; Validation cases median counts = 30.4; Training controls median = 22.2; Validation controls median = 14.6; rank sum p-values: Training vs. Validation cases p = 0.236; Training vs. Validation controls p = 0.126; Training cases vs. controls p = 0.719; Validation cases vs controls p = 0.058.

Supplementary Figure S10

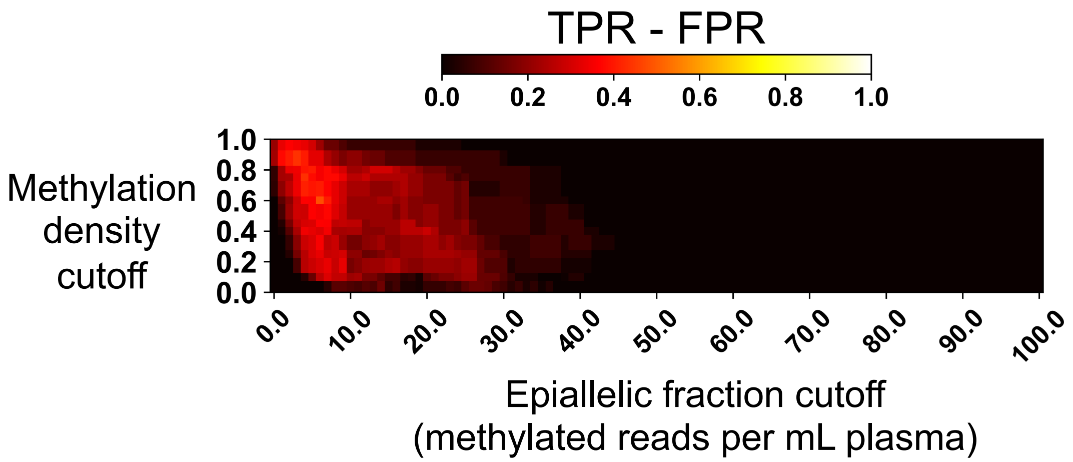

Supplementary Figure S10: EpiClass heatmap indicating the true and false positive rate differences for each combination of methylation density and epiallelic fraction cutoffs for identification of EOC (n=26) versus healthy control (n=41) plasma samples.

Supplementary Figure S11

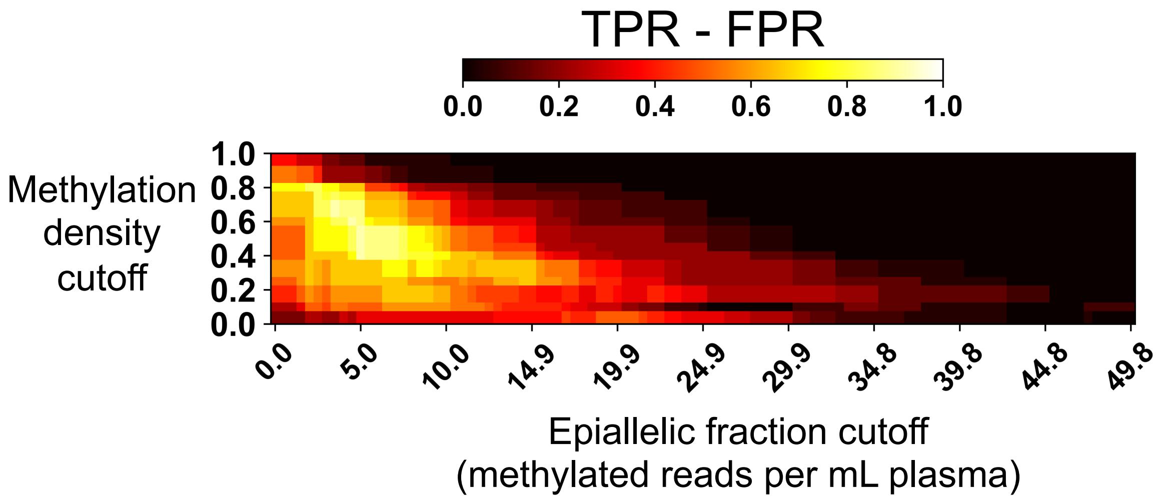

Supplementary Figure S11: EpiClass analysis of cfDNA DREAMing data from the validation cohort. **(A)** EpiClass heatmap indicating the true and false positive rate differences for each combination of methylation density and epiallelic fraction cutoffs for identification of EOC patient (n=24) versus healthy control (n=12) plasma samples of the second cohort.
